# Drs. Taliaferro & Noble

**Published:** 1884-07

**Authors:** 


### DRS. TALIAFERRO & NOBLE.

We had the pleasure, not many days since, of going through and inspecting the private infirmary of these gentlemen, situated at 170, 180 and 184 South Pryor street, Atlanta. We were surprised at what we saw. We had no idea that such an institution could be found in the South. This infirmary was established in the spring of 1881, and has grown steadily until now it includes three large two-story buildings fitted up in the most approved style. We found electric call bells in every room, placed convenient to the beds. The bath rooms are the most complete to be found anywhere, including hot, cold, shower and electrical baths. We were shown the conveniences and appliances for massage and electricity by the Weir Mitchell method. The batteries for applying electricity are the most complete we ever saw. It gives us pleasure to note the fact that these gentlemen are being abundantly rewarded for their efforts at establishing and maintaining a first-class infirmary where patients may go and receive not only the very best medical attention, but the luxuries of an elegant home as well.

